# Clinical outcomes and bone marker changes in postmenopausal women with dental implants: a one-year prospective study

**DOI:** 10.1186/s40729-025-00628-4

**Published:** 2025-05-24

**Authors:** Jung Min Cho, Namki Hong, Yumie Rhee, Wonse Park, Kyung Chul Oh, Yanggyung Seo, Hwangyu Lee, Hyeon-Gyu Jo, Yunji Shin, Jun-Young Kim

**Affiliations:** 1 Department of Oral and Maxillofacial Surgery and Oral Science Research Center, Yonsei University College of Dentistry, 50-1 Yonsei-Ro, Seodaemun-Gu, Seoul, 03722 Korea; 2 Department of Internal Medicine, Endocrine Research Institute, Yonsei University College of Medicine, Seoul, South Korea; 3 Department of Advanced General Dentistry, Yonsei University College of Dentistry, Seoul, South Korea; 4 Department of Prosthodontics, Yonsei University College of Dentistry, Seoul, South Korea; 5 Department of Oral Science Research Center, Yonsei University College of Dentistry, Seoul, South Korea; 6 Institute for Innovation in Digital Healthcare, Yonsei University Health System, Seoul, South Korea

**Keywords:** Osteoporosis, Osteopenia, Dental implants, Survival rate, Bone mineral density, Bone turnover marker

## Abstract

**Objectives:**

The feasibility of dental implants in patients with osteoporosis remains controversial, with limited prospective studies on quantitative changes in bone mineral density (BMD) and bone turnover markers (BTMs). This study assessed implant survival and clinical outcomes while evaluating systemic changes during 1 year of implant treatment.

**Materials and methods:**

Postmenopausal women requiring dental implants were enrolled at the Yonsei University Dental Hospital. BMD and BTMs were evaluated in collaboration with the endocrinology department. Participants were divided into two groups: Group A (T-score ≥ -2) and Group B (T-score < -2). All implants used in the study were surface-treated with hydroxyethyl piperazine ethane sulfonic acid (HEPES), and clinical, radiographic, and systemic parameters were monitored for over 1 year.

**Results:**

Between April 2022 and May 2024, 45 implants were placed in 36 patients (mean age: 68 years). Group A included 17 patients with 21 implants (mean age: 66 years), and Group B included 19 patients with 24 implants (mean age: 70 years). The cumulative survival rate was 100%. Resonance frequency analysis at 12 months revealed a mean implant stability tester value of 71.4 ± 5.52, indicating excellent osseointegration. Peri-implant bone loss averaged 0.54 ± 0.35 mm. No implant failures occurred, with stable plaque scores, probing depths, and bleeding upon probing. BMD and BTMs changes were minimal.

**Conclusions:**

Both groups achieved high implant survival and stable clinical outcomes. Systemic evaluations confirmed only minor changes in BMD and BTMs over 1 year. Larger multicenter studies are required to confirm the systemic safety of dental implants in patients with osteoporosis.

**Clinical relevance:**

Dental implants show excellent survival and stability in postmenopausal women with osteoporosis, with minimal impact on bone density and turnover—supporting their safe use in this population.

**Clinical trial registration:**

This study was prospectively registered at the Clinical Research Information Service of the National Research Institute of Health, Republic of Korea (KCT0007100). The registration details can be accessed at https://cris.nih.go.kr.

**Supplementary Information:**

The online version contains supplementary material available at 10.1186/s40729-025-00628-4.

## Introduction

Approximately 30% of women aged over 50 years experience osteoporosis [1, 2], with over 200 million cases reported globally [3]. In the European Union, 22 million women and 5.5 million men are affected annually, leading to 3.5 million fractures [4]. As global populations continue to age, the incidence of osteoporosis and related fractures are rising, posing significant health and economic burdens [5]. For instance, osteoporosis-related fractures in China are projected to reach 4.83 million cases by 2035, with associated costs increasing from $19.92 billion in 2035 to $25.43 billion by 2050 [6].

Osteoporosis is a systemic skeletal disorder characterized by reduced bone mass and deterioration of bone microarchitecture. These changes include a reduction in the number and thickness of trabeculae, disconnection of trabecular structures, cortical thinning, and increased bone porosity. These structural changes lead to weakened bone strength, heightened fragility, and an elevated risk of fracture [7, 8]. According to the World Health Organization (WHO), osteoporosis is defined as a skeletal disease involving a 25% reduction in bone mass, whereas osteopenia, considered a precursor of osteoporosis, is marked by a 10%–25% decrease in bone mineral density (BMD) [8]. Patients often receive periodic injections or oral medications that inhibit bone resorption and formation [9]. However, these treatments can also affect osseointegration, which is a critical determinant of the success of dental implants. Altered osseointegration not only increases the risk of implant failure but also the potential for medication-related osteonecrosis of the jaw (MRONJ) [10, 11]. Consequently, the medical history of osteoporosis and its treatment must be carefully evaluated before dental implant procedures, as both the disease and its therapies affect jawbone health [12].

Previous studies have recommended a 3-month drug holiday before and after dental implant placement in patients treated with bisphosphonates for over 3 years [13, 14]. However, these recommendations have evolved over time. The latest position paper by the American Association of Oral and Maxillofacial Surgeons highlights that the need for a drug holiday in patients with osteoporosis treated with bisphosphonates remains controversial [15].

In dental implant treatment, the potential for MRONJ underscores the importance of comprehensive dental care before initiating anti-resorptive drug (ARD) therapy. McGowan et al. emphasized that optimizing oral health and maintaining preventive dental care before beginning ARDs are essential [16]. A recent consensus statement indicates that dental implant treatment can be safely conducted while maintaining low-dose ARD therapy; however, it also underscores the need for further research [17, 18].

This study aimed to address the existing gaps in the literature by prospectively analyzing the clinical outcomes and changes in osteoporosis-related bone turnover markers (BTMs) in patients undergoing dental implant treatment. This study provides new scientific evidence by integrating BMD measurements into the evaluation. To date, no prospective studies have comprehensively examined these relationships.

## Materials and methods

### Study design

This single-center prospective study was conducted at the Department of Oral and Maxillofacial Surgery (OMFS), Yonsei University Dental Hospital, from April 2022 to May 2024. This study was approved by the Institutional Research Ethics Committee of Yonsei University College of Dentistry (IRB No. 2–2021-0116). All patients were thoroughly informed about the purpose and procedures of the study, and written informed consent was obtained prior to their participation. This study adhered to the ethical principles outlined in the Declaration of Helsinki for biomedical research involving human subjects. The manuscript was prepared in accordance with the STROBE guidelines for its design and reporting. It was also registered with the Clinical Research Information Service of the National Research Institute of Health, Republic of Korea (KCT0007100).

### Sample size calculation

The required sample size for the primary outcome variable (marginal bone loss) was calculated using the G*Power software (version 3.1.9.2; Heinrich-Heine-Universität Düsseldorf, Düsseldorf, Germany). Based on the study by Varga Jr. et al., the sample size calculation used a significance level (α) of 0.05, power (1 − β) of 0.8, and group means of 1.34 (standard deviation [SD] = 1.15) and 0.60 (SD = 0.36). The effect size was 0.8684. The total sample size was 36, considering a dropout rate of 10%, and a final sample size of 40 was assigned [19].

### Patient recruitment

Forty-three postmenopausal women were screened for eligibility based on inclusion and exclusion criteria (Table 1). Participants were provided with detailed verbal and written explanations of the study, its purpose, and procedures. Informed consent was obtained before participation. Screening included collection of demographic data, clinical and radiological assessments, BTM results, and BMD measurements at the hip, spine, and femoral neck. The patients were divided into two groups based on their lowest T-scores.

- Group A (T-score ≥ −2, n = 17)
- Group B (T-score < −2, n = 19)

**Table 1:** Inclusion and exclusion criteria for study participation

	Inclusion criteria
1	Provision of informed consent
2	Female sex, postmenopausal status and age ≥ 55 years
3	Need for implant in the maxilla or mandible
4	Absence or presence of osteopenia/osteoporosis (T-score > − 3) *Patients with T-scores < − 3 were excluded due to the urgency of treatment needs, as implant therapy could delay more critical interventions. This exclusion reflects ethical considerations to prioritize patient safety and appropriate care
	Exclusion criteria
1	Lack of ability to comply with the study procedures, as judged by the investigator
2	Known or suspected current malignancy
3	History of chemotherapy
4	History of radiation in the head and neck region
5	History of other metabolic bone diseases, for example Paget’s disease, hyperparathyroidism, fibrous dysplasia or osteomalacia
6	Medical history that makes implant insertion unfavorable
7	Uncontrolled diabetes mellitus, hypertension
8	Previous use of intravenous bisphosphonates or denosumab in 1 year
9	Previous use of oral bisphosphonates or denosumab in 1 year
10	Previous bone graft at other clinic or requiring extensive bone graft
11	Heavy smoking and alcohol consumption

**Fig. 1:**
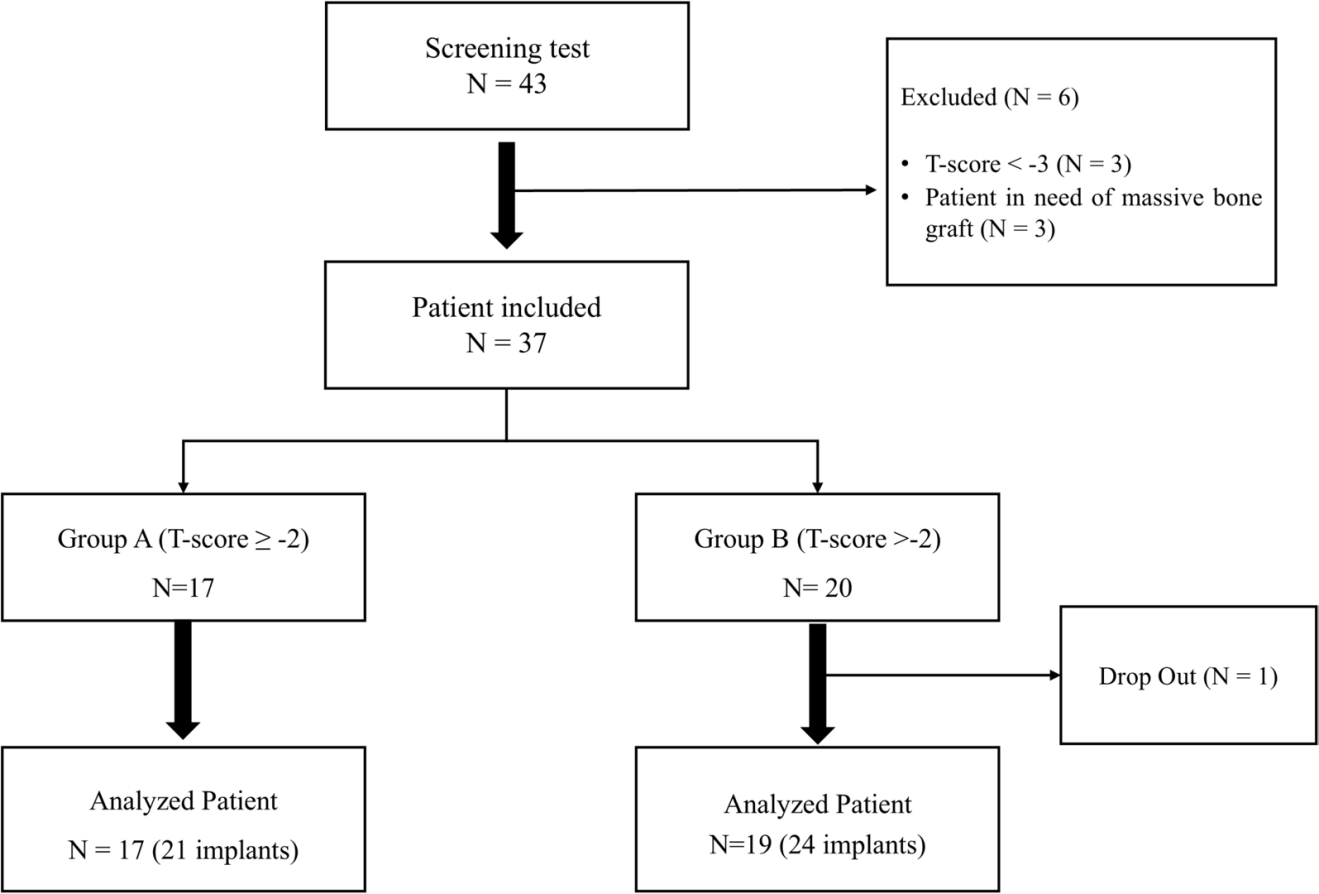
Flowchart depicting the patient screening and inclusion process

The threshold of − 2.0 was selected because it approximated the median T-score of the study population, allowing for a balanced group division. Although not aligned with the WHO osteoporosis classification, this threshold reflects the specific characteristics of the cohort. Three patients with T-scores < − 3 and three patients requiring additional bone grafts were excluded. Finally, a total of 37 patients were enrolled in this study (Fig. 1). Endocrinology specialists reviewed the BMD measurements and laboratory results to ensure comprehensive care.

### Study procedures

**Fig. 2:**
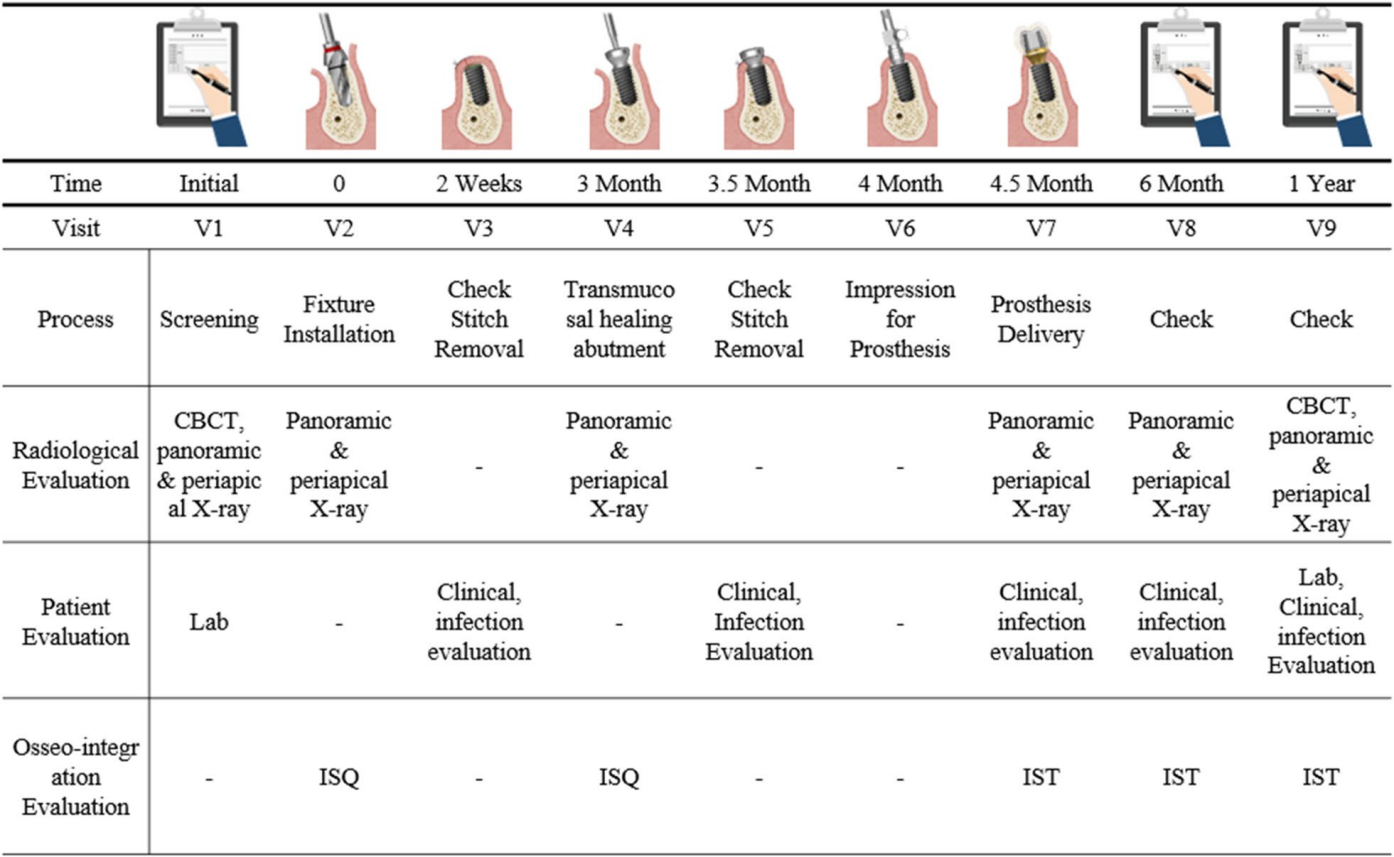
Illustration of study procedures and timeline. ISQ, implant stability quotient; IST, implant stability tester; V, visit

The participants attended nine scheduled visits over a 1-year study period (Fig. 2). During the first visit, radiographic evaluations were conducted using panoramic radiographs (RAYSCAN α, Ray Co. Ltd, Hwaseong-si, Korea) and cone-beam computed tomography (CBCT) scans (RAYSCAN α+, Ray Co. Ltd, Hwaseong-si, Korea) for implant treatment planning. Following the OMFS surgeons’ assessment of implant necessity and fulfillment of the inclusion criteria, endocrinological evaluations, including the BMD measurements and laboratory tests, were performed. Group B patients (T-score < − 2) received preoperative vitamin D supplementation to optimize bone health, ensuring ethical consideration for patient safety during the study, whereas Group A patients (T-score ≥ − 2) were advised to adopt dietary and lifestyle modifications.

A single experienced oral and maxillofacial surgeon performed all implant placement surgeries and second-stage procedures. All prosthodontic procedures, including impression-taking and prosthesis delivery, were carried out by a single prosthodontist who is a faculty member in the Department of Prosthodontics. Overall treatment planning, postoperative follow-up, and outcome evaluations were conducted within the Department of Oral and Maxillofacial Surgery, following a standardized interdisciplinary protocol to ensure procedural consistency.

During the second visit, the implants were placed under local anesthesia. After the crestal incision, surface treated with hydroxyethyl piperazine ethane sulfonic acid (HEPES) implants (Osstem SOI Implant, Osstem Implant, Seoul, South Korea) were inserted into the alveolar bone. Initial implant stability was measured using the implant stability quotient (ISQ) and periapical radiographs (KODAK Digital X-ray Specimen software, Carestream RVG 2200 intraoral X-ray system with RVG 6200 sensor, Carestream Dental, Rochester, NY, USA) and panoramic views were obtained for baseline records. Postoperatively, the patients were prescribed antibiotics and anti-inflammatory medications.

At visit 3 (2 weeks post-surgery), the sutures were removed, and any signs of infection or complications were evaluated. The patients received additional instructions for chlorhexidine mouth rinses or further medications as needed. Visit 4, conducted 3 months post-surgery, involved a second-stage procedure in which healing abutments were connected after a small buccal flap incision. Implant stability was assessed using the ISQ and implant stability tester (IST) values to confirm successful osseointegration. Patients were referred to the prosthodontics department for crown fabrication.

Visit 5 included impression-taking for the prosthetic crowns and visit 6 involved the final prosthetic placement where the implant crowns were installed. During these visits, clinical evaluations including probing depth, plaque score, and bleeding on probing were performed. At visit 7, the implant crowns were installed, completing the prosthetic rehabilitation process. Follow-up evaluations (visits 8 and 9) were conducted 6 months and 1 year post-surgery, respectively, involving IST value measurements, radiography, and peri-implant clinical assessments. At visit 9, follow-up CBCT imaging and endocrinological evaluations were performed to assess systemic changes in BMD and the laboratory parameters. Implant success rates were evaluated using Buser’s criteria [20].

### Primary outcome

**Fig. 3:**
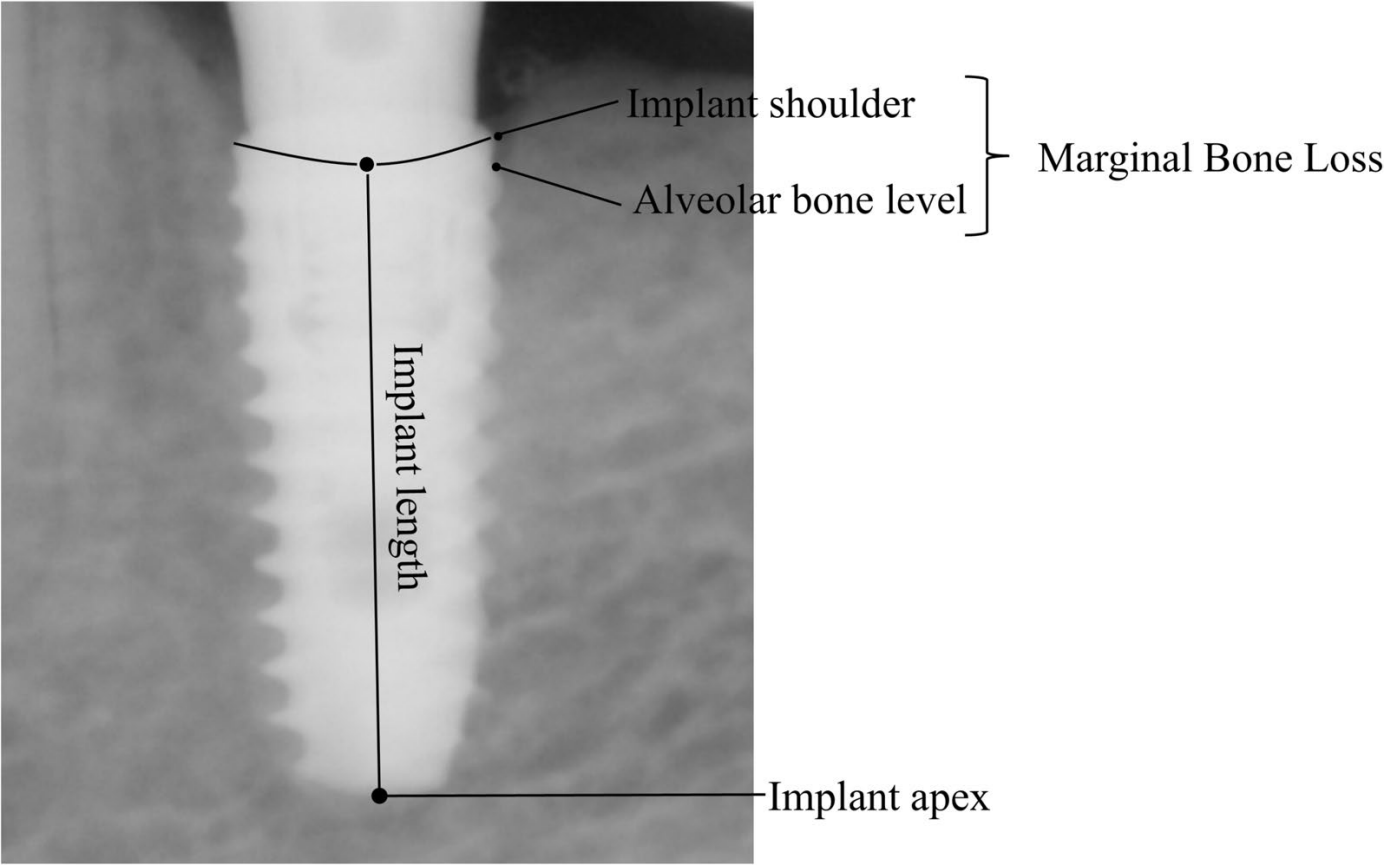
Measurement of Peri-Implant Marginal Bone Loss

The primary outcome was marginal bone loss (MBL), which was assessed using a picture archiving and communication system (Zetta PACS, TaeYoung Soft, Kyunggi-do, South Korea). Radiographic images, including periapical radiographs and CBCT scans, were used to measure bone resorption on the mesial, distal, buccal, and lingual/palatal sides of the implant. (The MBL refers to the amount of bone resorption below the implant thread (Fig. 3). Radiographs were individually calibrated to correct for size errors using the length of the implant and the distance between the threads.

MBL was assessed at four sites around the implant: mesial, distal, buccal, and lingual. The mesial and distal measurements were obtained using periapical radiographs, while the buccal and lingual measurements were conducted using CBCT. Marginal bone loss (MBL) was measured twice at two different time points by a single calibrated examiner using standardized radiographic reference points. The first measurement of the two measurements was used in the final analysis.

### Secondary outcome

This study aimed to evaluate the success of implant treatment in all patients by assessing osseointegration, pocket probing depth, bleeding on probing, and clinical attachment loss. Osseointegration was monitored at visits 2, 4, 7, 8, and 9 using the ISQ (Osstell AB, Sweden) and an IST (Anycheck, Neobiotech Co., Ltd, South Korea). These measurements provided data on implant success rates and facilitated group comparisons. Additionally, clinical parameters, such as pocket probing depth, bleeding on probing, and clinical attachment loss, were examined to ensure compliance with the established implant success criteria.

Systemic markers were analyzed to evaluate the changes in bone metabolism. Bone turnover markers include the C-terminal telopeptide of type I collagen (CTx), a marker of bone resorption, and the procollagen type I N-terminal propeptide (P1NP), a marker of bone formation. BMD was measured in the lumbar spine, femoral neck, and total hip using dual-energy X-ray absorptiometry. Parathyroid hormone (PTH) and vitamin D levels were assessed to monitor calcium homeostasis and nutritional status. To evaluate the presence of osteoporotic fractures, whole-spine radiographs were obtained at baseline (visit 1) and the final follow-up (visit 9). These radiographs aimed to identify vertebral fractures that could affect systemic outcomes or treatment evaluation.

### Statistical analysis

Statistical analyses were performed using SPSS Statistics for Windows, version 28.0 (IBM Corp., Armonk, NY). The objective of this study was to evaluate differences in clinical, laboratory, and BMD outcomes between Groups A and B over a 1-year study period, based on a two-sided hypothesis.

The normality of the data distribution was assessed using Shapiro–Wilk and Kolmogorov–Smirnov tests. For normally distributed data, the paired *t*-test was applied to compare measurements before implant placement and 1 year post-implantation. Statistical significance was set at *p* < 0.05.

MBL measurements were performed twice, two weeks apart, by a single examiner (X1 and X2). Intra-examiner reliability was assessed using the intra-class correlation coefficient (ICC), while systemic and random errors between the two measurements were calculated using the Dahlberg formula. From this study, data from the first measurement (X1) were used [21]. Normal distribution of data was established using the Shapiro–Wilk and Kolmogorov–Smirnov tests.

## Results

### Patient sample

A total of 45 implants were placed in 36 patients with an average age of 68 years (range: 54–86 years). In Group A, 21 implants were placed in 17 patients with an average age of 66 years (range: 54–79 years), whereas in Group B, 24 implants were placed in 19 patients with an average age of 70 years (range: 56–86 years). All patients were non-smokers and underwent treatment under local anesthesia with prescribed antibiotics and anti-inflammatory medications. Baseline demographics and characteristics of the study groups, including statistical comparisons, are presented in Table 2. Additional implant-related information is summarized in Table 3. In both groups, all implants were bone-level types and restored with single crowns.

**Table 2:** Demographics and Baseline Clinical Data of Study Participants

	Group A (n = 17) T-score > − 2	Group B (n = 19) T-score ≤ − 2	Total (n = 36)	*p*-value
Characteristic				
Sex	Female	Female	Female	
Age (mean ± SD)	65.59 ± 7.68	70.00 ± 8.08	67.92 ± 8.10	0.100
Smokers (n, %)	0 (0%)	0 (0%)	0 (0%)	-
Hypertension (n, %)	6 (35.3%)	11 (57.9%)	17 (47.2%)	0.354
Diabetes (n, %)	1 (5.9%)	5 (26.3%)	6 (16.7%)	0.182
History of steroid use (n, %)	0 (0%)	0 (0%)	0 (0%)	-
T-score (mean ± SD)				
Spine L1–L4	−1.20 ± 0.25	−2.0 ± 0.29	−1.62 ± 0.65	< 0.001
Femoral neck	−1.08 ± 0.31	−2.41 ± 0.30	−1.78 ± 0.87	< 0.001
Total hip	−0.39 ± 0.54	−1.38 ± 0.36	−0.91 ± 0.83	< 0.001
Therapy				
Implant system	Osstem SOI Implant (bone-level)			
Surface coating	hydroxyethyl piperazine ethane sulfonic acid (HEPES)			
Implant number	21	24	45	

SD, standard deviation

**Table 3:** Implant information

	Group A (n = 21)	Group B (n = 24)	Total (n = 45)
Implant location (Jaw)			
Maxilla (%)	14 (66.7%)	9 (37.5%)	23 (51.5%)
Mandible (%)	7 (33.3%)	15 (62.5%)	22 (48.5%)
Type of prosthesis			
Single crown (%)	21 (100%)	24 (100%)	45 (100%)
Implant macro-design			
Bone level (%)	21(100%)	24(100%)	45(100%)

### MBL

**Fig. 4:**
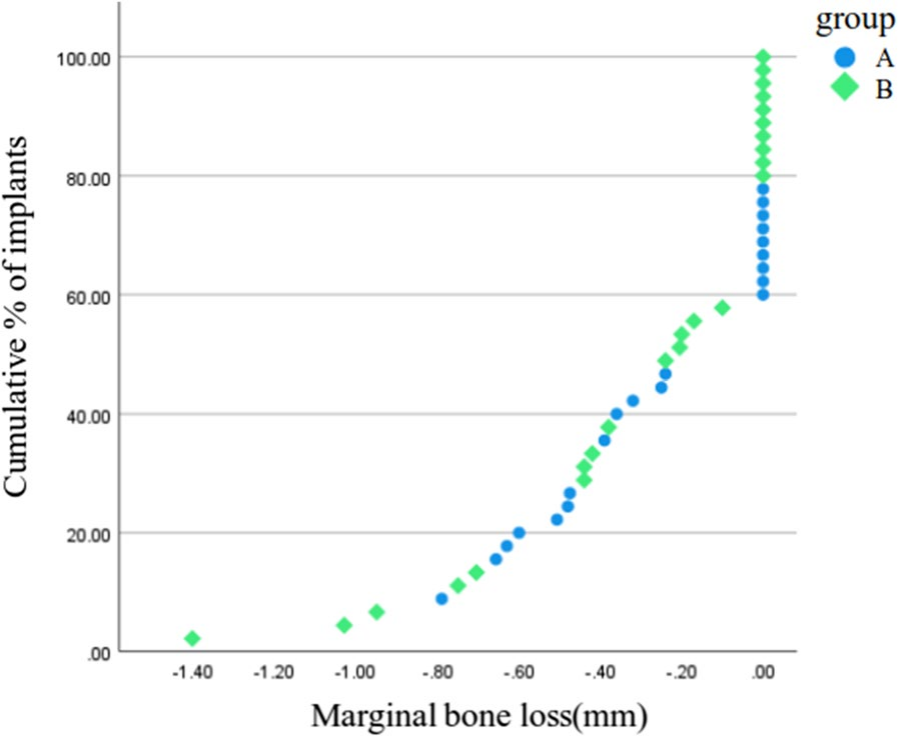
Comparison of 1-year marginal bone loss between group A and B

MBL was evaluated at the final follow-up (visit 9) using periapical radiographs and CBCT scans. Bone loss was measured from the top of the implant fixture, with any bone gain recorded as zero. Table 4 summarizes the mean MBL by jaw location; in Group A, it was 0.25 mm in the maxilla and 0.30 mm in the mandible; in Group B, the corresponding values were 0.37 mm and 0.27 mm. No statistically significant differences were observed between groups or jaw locations (*p* > 0.05). Cumulative MBL values are illustrated in Fig. 4.

**Table 4:** Comparison of Marginal Bone Loss and Reliability analysis of intra examiner measurements of Marginal Bone Loss between Group A and B

	Group A (N = 21)		Group B (N = 24)	
MBL	Mx. (N = 14)	Mn. (N = 7)	Mx. (N = 9)	Mn. (N = 15)
Mean	0.25	0.30	0.37	0.27
SD	0.25	0.33	0.43	0.38
p-value*	0.40	0.84	0.40	0.84

	Intra-examiner reliability (ICC)				
Group (N)	Sum of variables (X1–X2)^2^	Random error (Dahlberg)	ICC	95% Cl (lower)	95% Cl (higher)
A(21)	0.007	0.017	0.9907	0.985	0.998
B(24)	0.018	0.025	0.996	0.993	0.999
Total(45)	0.025	0.022	0.99	0.994	0.998

X1, first measurement; X2, second measurement

*Paired t-tests were used for statistical comparisons

The systematic error measured using the Dahlberg formula was remarkably low. The ICC for intra-examiner reliability exceeded 0.9, indicating acceptable consistency in landmark digitization by the same examiner across multiple assessments (Table 4). Intra-examiner reliability was assessed using the Dahlberg formula and the intraclass correlation coefficient (ICC). ICC values were 0.9907 for Group A and 0.996 for Group B, with an overall study ICC of 0.99—indicating acceptable reproducibility. The calculated Dahlberg errors were below 0.03 mm in all groups, confirming high consistency of the repeated MBL measurements.

**Table 5:** Comparison of implant stability quotient and implant stability tester values between groups A and B at different time points

ISQ/IST values*	Group A (N = 21)						Group B (N = 24)					
	Mx. (N = 14)			Mn. (N = 7)			Mx. (N = 9)			Mn. (N = 15)		
	V2	V4	V9	V2	V4	V9	V2	V4	V9	V2	V4	V9
Mean	69.14	75.07	72.86	78.71	81.43	71.43	75.22	74.56	73.89	79.93	76.80	71.80
SD	9.20	4.97	5.71	5.50	5.22	5.16	5.91	6.41	5.47	6.66	6.51	6.32
p-value†﻿	0.09	0.83	0.67	0.68	0.12	0.89	0.09	0.83	0.67	0.68	0.12	0.89

^*^ISQ was measured at V2, and IST was measured at V4 and V9

ISQ, implant stability quotient; IST, implant stability tester; V, visit; SD, standard deviation

^†^ Paired t-tests were used for statistical comparisons

### Osseointegration

Osseointegration was assessed at multiple time points using Resonance Frequency Analysis (RFA) and implant stability tester (IST) values. Table 5 presents implant stability measurements stratified by jaw location and study group. As shown in Table 5, both groups achieved acceptable initial and final stability values, with no statistically significant differences throughout the observation period. Across all visits, implants placed in the mandible consistently demonstrated higher stability values than maxillary implants in both groups.

At visit 2, the mean ISQ in Group A was 78.71 for mandibular implants and 69.14 for maxillary implants. Similarly, in Group B, mandibular implants showed a mean ISQ of 79.93, while maxillary implants showed 75.22. This pattern was maintained at subsequent visits using IST measurements: at visit 4, Group A showed 81.43 (mandible) vs. 75.07 (maxilla), and Group B showed 76.80 (mandible) vs. 74.56 (maxilla).

At the final follow-up (visit 9), all subgroups maintained mean IST values above 70, indicating adequate implant stability. No statistically significant differences in ISQ or IST values were found between Groups A and B (*p* > 0.05).

### Peri-implant health evaluation

Peri-implant health was assessed at visits 7, 8, and 9. Table 6 summarizes the clinical parameters, including probing pocket depth (PPD), bleeding on probing (BOP), and presence of plaque.

**Table 6:** Comparison of peri-implant clinical parameters between groups A and B at different time points

	Group A			Group B		
	V7	V8	V9	V7	V8	V9
PPD						
Mean (Mx/Mn)	3.00/3.42	3.50/4.00	3.00/2.43	4.22/2.87	3.22/2.67	3.56/2.40
SD (Mx/Mn)	1.24/1.81	1.40/1.63	0.68/1.51	0.97/1.69	1.20/1.35	1.51/1.06
p-value* (Mx/Mn)	0.21/0.49	0.63/0.06	0.24/0.96	0.21/0.49	0.63/0.06	0.24/0.96
BOP (N, %)†	8 (38.1%)	11 (52.4%)	8 (38.1%)	6 (25.0%)	9 (36.0%)	10 (41.7%)
Plaque (N, %)†	0	3 (14.3%)	5 (23.8%)	0	4 (16.7%)	4 (16.7%)

^*^ Paired *t*-tests were used for statistical comparisons

^†^ BOP was considered if any implant site showed bleeding upon probing. Plaques were counted if any implant site exhibited a visible plaque

SD, standard deviation; PPD, probing pocket depth; BOP, bleeding on probing; V, visit

PPD values were reported separately for the maxilla and mandible. At visit 9, Group A showed a mean PPD of 3.00 mm in the maxilla and 2.43 mm in the mandible, while Group B showed 3.56 and 2.40 mm, respectively. Across all time points, mandibular implants tended to exhibit slightly lower PPD values compared to maxillary implants. However, no statistically significant differences were observed between groups (*p* > 0.05).

The prevalence of BOP ranged from 25.0% to 52.4% across groups and time points, while visible plaque was observed in up to 23.8% of cases. Overall, peri-implant soft tissue health remained stable, with no statistically significant differences between groups.

### BMD and BTM outcomes

Changes in BMD and BTMs between baseline (visit 1) and final follow-up (visit 9) are summarized in Table 7. The mean change in overall T-score was − 0.16 ± 0.35 in Group A and − 0.01 ± 0.31 in Group B, with no significant difference between the groups (*p* = 0.244). Similarly, regional BMD measurements (spine, femoral neck, and total hip) showed minimal changes without statistical significance (*p* > 0.05 for all).

**Table 7:** Comparison of 1-year Systemic Outcomes in Groups A and B

	Group A (n = 17)	Group B (n = 19)	Total (n = 36)	*p*-value*
△T-score	− 0.16 ± 0.35	− 0.01 ± 0.31	− 0.08 ± 0.33	0.244
Spine L1–L4	− 0.05 ± 0.41	0.12 ± 0.36	0.04 ± 0.39	0.186
Femoral neck	− 0.14 ± 0.30	− 0.04 ± 0.27	− 0.09 ± 0.29	0.300
Total hip	− 0.08 ± 0.27	0.02 ± 0.27	− 0.03 ± 0.27	0.175
△ PTH	2.82 ± 10.96	2.59 ± 17.65	2.70 ± 14.67	0.900
△ 25(OH) Vit. D	1.17 ± 12.89	11.58 ± 12.62	6.67 ± 13.63	0.015†
△ CTx	− 0.07 ± 0.18	− 0.08 ± 0.19	− 0.08 ± 0.18	0.531
△ P1 NP	− 1.15 ± 19.05	− 14.39 ± 16.96	− 8.14 ± 17.26	0.033†

Data are presented as mean ± SD

△ values represent changes between baseline (visit 1) and one year post-operation (visit 9): absolute value

Negative values represent an increase in the measurement values at visit 9

^*^ Paired *t*-tests were used for statistical comparisons

^†^ Significant differences (*p* < 0.05)

MBL, marginal bone loss; ISQ, implant stability quotient; IST, implant stability tester; PPD, probing pocket depth; PTH, parathyroid hormone; 25(OH) Vit. D, vitamin D; CTx, C-terminal telopeptide of type I collagen; P1NP, procollagen type I N-terminal propeptide; SD, standard deviation

Among BTMs, changes in PTH and CTx were not significantly different between groups. However, Group B showed a significantly greater increase in serum 25(OH) vitamin D levels (*p* = 0.015), consistent with preoperative supplementation. Additionally, Group B exhibited a significantly greater decrease in P1NP levels compared to Group A (*p* = 0.033). No osteoporotic fractures-related complications were observed on whole-spine radiographs at either time point.

## Discussion

Systemic health is essential for a successful implant integration. However, as the global population ages and natural teeth are retained longer, the demographics of implant patients have shifted to include individuals with various systemic conditions, such as osteoporosis. Osteoporosis, which is characterized by reduced BMD and increased fracture risk [22], presents new challenges for implant therapy, necessitating the reassessment of traditional principles.

This study categorized patients based on a T-score threshold of −2.0 to facilitate group comparisons. Although the WHO classifies T-scores into three categories (− 1.0 to − 2.5 for osteopenia and < − 2.5 for osteoporosis) [23], Korean epidemiological data highlights a high prevalence of both osteopenia and osteoporosis among postmenopausal women, particularly those aged over 60 years [24]. Biological changes associated with osteoporosis, such as deteriorating bone microarchitecture, have historically raised concerns regarding osseointegration and implant failure. However, recent reviews by Mombelli et al. and Bornstein et al. suggested that the impact of osteoporosis on implant success may be less significant than previously assumed [25].

Concerns regarding osteonecrosis of the jaw (ONJ), especially in bisphosphonate-treated patients, have influenced implant treatment protocols. Although bisphosphonates are essential for managing osteoporosis, questions have been raised regarding their implications in implant procedures. However, emerging studies have indicated that careful monitoring and management can mitigate these risks, making implant placement feasible in bisphosphonate-treated patients [26, 27].

In this study, MBL was closely monitored as the primary outcome. Overall, MBL showed minimal variation across groups and jaw locations. At the 1-year follow-up, mean MBL ranged from 0.25 to 0.37 mm depending on group and jaw (Table 5), with no significant differences observed. Importantly, MBL was slightly higher in maxillary implants than in mandibular implants within Group B, although the clinical relevance of this difference remains limited due to the small absolute values. The observed MBL remained within acceptable limits, with an average of 0.54 mm at 1 year, consistent with the literature stating that MBL within 2 mm during the first year of functional loading is considered normal [28, 29]. Intra-examiner measurement reproducibility was excellent, with ICC values exceeding 0.99 and Dahlberg errors below 0.03 mm, ensuring the robustness of radiographic assessment.

Secondary outcomes included clinical parameters such as ISQ and IST values, PPD, BOP, and plaque evaluation. The ISQ and IST values, which reflect osseointegration, were consistently higher in mandibular implants compared to maxillary implants across both groups, at all time points (Table 4). Despite these anatomical differences, all implants maintained values above the clinical stability threshold, with no statistically significant differences between groups. These findings suggest that while anatomical site may affect absolute stability values, it does not compromise clinical osseointegration in this population. Additionally, all measured parameters showed no significant intergroup differences, indicating that implants can achieve similar stability in patients with and without osteoporosis. This observation aligns with findings from animal studies that demonstrated comparable ISQ and IST values in osteoporotic and healthy bone conditions [30, 31].

RFA values, typically expected to increase over time as osseointegration strengthens, can occasionally decrease owing to factors such as the length of the implant superstructure, as noted in the study by Al-Jetaily and Al-Dosari [32]. The variations in ISQ in this study were attributed to anatomical differences in the oral cavity and measurement height from the healing abutment to the prosthesis placement. Importantly, none of the ISQs fell below 60, the threshold for implant failure, suggesting that stability was maintained throughout the study.

Soft tissue health was well maintained in all patients. Probing pocket depth (PPD) values were generally slightly lower in mandibular implants compared to maxillary sites, particularly at visit 9 (e.g., 2.43 vs. 3.00 mm in Group A; 2.40 vs. 3.56 mm in Group B) (Table 6). Bleeding on probing (BOP) and visible plaque accumulation were also within acceptable clinical ranges, with no significant differences between the groups. This suggests that periodontal health around implants can be effectively maintained in both patients with and without osteoporosis. These findings align with previous studies, highlighting the critical role of regular dental care and proper hygiene management in ensuring the success of implant treatment, even in patients with systemic conditions [33].

Patients with osteoporosis often exhibit deteriorating bone microarchitecture and BMD. Although numerous studies have examined the effects of various therapies for patients with osteoporosis, there are limited data on BMD changes in patients who have not initiated ARD therapy during implant treatment. In this study, ARD therapy was not initiated for 1 year to avoid the potential risk of ONJ, which can be associated with these medications. Under these specific conditions, Group A showed a T-score change of − 0.16, and Group B showed a change of − 0.01, with no significant differences between the two groups. These findings suggest that, in the absence of ARD therapy, BMD remained relatively stable during implant treatment. However, this outcome was specific to the conditions in this study and should not be generalized to all patients with osteoporosis, particularly those undergoing ARD therapy. While marginal bone loss and implant stability were assessed as primary clinical outcomes, the broader significance of this study lies in demonstrating that implant treatment did not result in systemic deterioration of bone health in osteoporotic patients. Given the ongoing concerns surrounding implant placement in individual at risk of medication-related osteonecrosis of the jaw (MRONJ), our findings suggest that, with proper management, implant therapy may be safely considered in elderly patients with osteoporosis. These results contribute to the current understanding of implant safety in this increasingly prevalent elderly population. In addition, Group B received preoperative vitamin D supplementation due to lower baseline T-scores as an ethical precaution. While this difference in management may raise concerns regarding potential effects on systemic outcomes, prior randomized controlled trials have shown that vitamin D supplementation does not significantly alter bone turnover markers or bone mineral density in individuals without severe deficiency [34, 35]. In our study, baseline 25(OH) Vit. D levels were above the clinical deficiency threshold, suggesting that the observed differences were unlikely to be driven by supplementation alone. Nevertheless, this variation in group management is acknowledged as a limitation, and future research should consider aim to control vitamin D status more rigorously at baseline.

This study excluded patients with severe osteoporosis (T-score < − 3.0) and those requiring bone grafting procedures. Therefore, these findings are applicable primarily to patients with mild-to-moderate osteoporosis or those with sufficient bone quality for direct implant placement. Further studies, including patients with more severe conditions or those requiring bone augmentation, are needed to generalize these results.

This study has some limitations that warrant consideration. First, the relatively small sample size limited the generalizability of our findings. Additionally, the limited sample size and variation in patient background factors, such as age and comorbidities, constrained our ability to perform multivariate statistical adjustments. While baseline characteristics between groups, including age and systemic health conditions, were not significantly different (Table 2), residual confounding cannot be entirely ruled out. Therefore, the study findings should be interpreted within the scope of an exploratory investigation. Future studies with larger and more diverse populations should incorporate stratified analyses or regression models to validate these observations more robustly. Larger multicenter studies with expanded sample sizes are required to confirm the outcomes observed in this study. In addition, a longer follow-up period would provide more robust data on the long-term success and systemic effects of implant treatments in patients with osteoporosis. Second, the study was unable to recruit patients strictly adhering to the WHO classification for osteoporosis (T-score < − 2.5) and osteopenia (T-score − 1 to − 2.5) owing to the distribution of the population within the recruitment pool. Although this limitation reflects the demographic realities of the patient population, it highlights the need for future studies to explore larger and more diverse cohorts to better assess outcomes across all osteoporosis classifications. In addition to the limited sample size and short follow-up duration, several procedural factors may have influenced the outcomes observed. Variations in drilling technique, native bone quality at the implant site, and prosthodontic procedures (e.g., timing of loading, type of prosthesis, occlusal design) were not standardized across all cases. These uncontrolled variables may have impacted marginal bone loss and implant stability, independent of systemic bone health. Further investigations should aim to control procedural variability and extend follow-up duration to strengthen the clinical applicability of findings in osteoporotic patients.

## Conclusion

Despite the limitations of this study, including the small sample size, short follow-up period, and procedural variability, dental implant treatment appeared to yield stable clinical and systemic outcomes in postmenopausal women with mild-to-moderate osteoporosis. No significant differences were observed between groups in marginal bone loss, probing depth, or bone metabolism over the 1-year follow-up period. These preliminary findings suggest that implant therapy may be viable for this population, though caution is advised in interpreting these results. Further long-term and controlled studies are warranted to validate these results, particularly in patients with severe osteoporosis or compromised bone conditions.

## Supplementary Information

Additional file 1.

## Data Availability

No datasets were generated or analysed during the current study.

## Abbreviations

BMD: Bone mineral density
BTM: Bone turnover marker
MRONJ: Medication-related osteonecrosis of the jaw
ARD: Anti-resorptive drug
OMFS: Oral and Maxillofacial Surgery
HEPES: Hydroxyethyl piperazine ethane sulfonic acid
ISQ: Implant stability quotient
IST: Implant stability tester
MBL: Marginal bone loss
CTx: C-terminal telopeptide of type I collagen
P1NP: Procollagen type I N-terminal propeptide
PTH: Parathyroid hormone
PPD: Probing pocket depth
BOP: Bleeding on probing
